# The impact of childhood trauma on Adolescent Depressive Symptoms: the Chain Mediating role of borderline personality traits and self-control

**DOI:** 10.1186/s12888-024-05829-6

**Published:** 2024-05-21

**Authors:** Yingyan Zhong, Qianying Hu, Jianhua Chen, Yuting Li, Rumeng Chen, Yan Li, Enzhao Cong, Yifeng Xu

**Affiliations:** 1 Shanghai Mental Health Center, Shanghai Jiao Tong University School of Medicine, Shanghai, China; 2 School of Medicine, Shanghai Tenth People’s Hospital, Tongji University, Shanghai, China

**Keywords:** Childhood trauma, Depressive symptoms, Borderline personality traits, Self-control, Adolescent

## Abstract

**Background:**

The adolescent depression associated with childhood trauma has been confirmed, but the underlying mechanisms remain unclear. This study aims to explore the chain-mediated role of borderline personality traits and self-control in the relationship between childhood trauma and adolescent depression.

**Methods:**

A cross-sectional study was conducted on 2,664 students from a senior high school through online questionnaires from October to December 2022 in Henan, China. Childhood Trauma Questionnaire-Short Form, Borderline Personality Dimension of Personality Diagnostic Questionnaire-4, Self-Control Scale, and Children’s Depression Inventory were used to measure childhood trauma, borderline personality traits, and self-control.

**Results:**

The prevalence of depression in adolescents was 21.17%, while the prevalence of borderline personality was 12.00%. childhood trauma (*r* = 0.50, *p* < 0.001) and borderline personality traits (*r* = 0.60, *p* < 0.001) were positively correlated with adolescent depressive symptoms, while self-control was negatively correlated with depressive symptoms (*r* = − 0.50, *p* < 0.001). Borderline personality traits and Self-control both play a mediating role in childhood trauma and depressive symptoms, and the mediating effect values are 0.116 (95%CI = [0.098, 0.137]), and 0.022 (95%CI = [0.012, 0.032]) respectively. The chain mediating effect of borderline personality traits and self-control on the relationship between childhood trauma and depressive symptoms was significant (effect value: 0.034, 95%CI = [0.028, 0.042]).

**Conclusions:**

Childhood trauma can predict depressive symptoms in adolescents due to the formation of borderline personality traits and the reduction of self-control. These findings are important for understanding the formation of personality traits, self-control abilities and coping strategies shaped by traumatic experiences in adolescents.

## Introduction

Depression is a multifactorial disorder composed of behavioral-cognitive-social-biological symptoms characterized by depressed mood or loss of interest/pleasure, which collectively lead to a reduced ability to derive reinforcement from one’s environment, ultimately resulting in challenges in daily functioning [1, 2]. The prevalence of adolescent depressive symptoms has been a continuous increase worldwide, rising from 24% between 2001 and 2010 to 37% between 2011 and 2020 [3]. During the COVID-19 period (2021) in China, the prevalence of depressive symptoms in adolescents surged to a peak of 55.67% [4], before receding to 24.24% in 2022 [5]. The presence of depressive symptoms during adolescence can increase the likelihood of future functional impairments and psychological disorders [6]. Furthermore, depressive symptoms are also the most robust correlators and predictors of suicidal ideation [7].

Risk factors for depressive symptoms have been studied, including personality traits, coping strategies and negative life events [8]. Childhood trauma is a severe negative life event. Different types of childhood trauma often occur simultaneously [9, 10]. Childhood trauma significantly increases the risk of developing major depressive disorder in adulthood and links to a higher likelihood of chronic or recurrent depressive symptoms [11, 12]. Based on prior research, any type of childhood trauma has been linked to over a twofold increase in the risk of depression in adulthood [13]. Hence, childhood trauma is assumed to predict depressive symptoms.

Borderline personality disorder (BPD) mainly shows disturbances in the regulation of emotions, feelings of paranoia triggered by stress, and a sense of detachment from reality, which have a negative impact on depressive disorders [14]. There is a strong link between borderline personality disorders and depressive symptoms [15, 16]. Adolescents exposed to childhood trauma are more likely to develop borderline personality traits, which are prone to the manifestation of borderline personality disorder [17–19]. Previous research indicated a significant correlation between childhood trauma and BPD [17, 20], and childhood trauma is recognized as an important cause of BPD [21]. Adolescents with BPD had experienced some forms of childhood trauma, especially emotional abuse and neglect, which was associated with more depressive symptoms [22]. Since children and adolescents should not be easily diagnosed with borderline personality disorder [23], research should be done on the development process of borderline personality traits in adolescents [24]. Childhood trauma, especially emotional and sexual abuse was related to more borderline personality traits and depressive symptoms in adolescence and early adulthood [25]. Moreover, among individuals experiencing depressive symptoms, there was a substantial mediating effect of childhood trauma on suicide attempts, mediated by borderline personality traits [26]. Therefore, we assume borderline personality traits play a mediating role between childhood trauma and depressive symptoms.

Self-control is the ability to align thoughts, emotions, and behaviors with long-term valued objectives even in the presence of temporarily more attractive alternatives [27]. But childhood trauma impairs the function of self-control [28]. Self-control was also negatively related to depressive symptoms in adolescents [29]. In addition, high self-control could be a protective factor for adolescent depressive symptoms through adjusting perceived stress [30]. Based on the research mentioned above, we reasonably assume self-control may be the mediator between childhood trauma and depressive symptoms.

Depressive symptoms are associated with negative life events, personality traits and coping strategies [8]. One of the most obvious characteristics of both borderline personality and depression is a reduction in self-control [31, 32]. Poor self-control in childhood also predicts borderline personality traits in adolescents [33]. Thus, the degree of borderline personality and self-control affect each other in adolescents. Therefore, this study assumes the chain-mediated role of borderline personality traits and self-control was played in in the effect of childhood trauma on adolescent depressive symptoms.

In summary, this study aimed to explore the relationship between childhood trauma, borderline personality traits, self-control, and adolescent depressive symptoms. We hypothesize that (1) Childhood trauma and borderline personality traits are positively associated with depressive symptoms, but self-control is negatively associated with depressive symptoms; (2) Borderline personality traits and self-control have mediating effects on the relationship between childhood trauma and adolescent depressive symptoms respectively. (3) Borderline personality traits and self-control play a chain mediating role in the relationship between childhood trauma and adolescent depressive symptoms.

## Methods

### Participants and procedure

The research took place from November 17, 2021, to December 11, 2021. The adolescents were from a high school (Grade 10 to Grade 12) in Henan Province, China. The school psychologists and class teachers from this school administered a survey via an online platform. At the beginning of the survey, an informed consent form was presented. Participants who selected “Agree” proceeded to the survey, and those who selected “Disagree” exited the survey automatically. 2,694 adolescents completed the survey. Repeated submissions (*n* = 5) were identified through student numbers and eliminated. Submissions with a completion time of more than 3 standard deviations (*n* = 25) were deemed invalid and eliminated. Hence, a final sample of 2,664 adolescents (valid response rate: 98.89%) was included. The study was approved by the Ethics Committee of Shanghai Mental Health Center, China (Ethics Approval Number: 2021-11).

### Measures

#### Childhood trauma

The Childhood Trauma Questionnaire-Short Form (CTQ-SF) was used to measure adolescents’ trauma experiences in childhood [34]. It has been normalized in China with high reliability and validity, the Cronbach’s α coefficient of which was 0.81, and the criterion-related validity coefficient of which was 0.61 [35]. The scale has 28 items, each rated on a 5-point Likert scale, ranging from 1 (never true) to 5 (very often). A higher score represents more perceived traumatic experiences in childhood. In the current sample, Cronbach’s α was 0.577.

#### Borderline personality traits

The Borderline personality dimension of the Chinese revision of Personality Diagnostic Questionnaire-4 (PDQ-4) was used to assess borderline personality traits in adolescents [36, 37]. The subscale of borderline personality is made up of 9 items, including Items 6, 17, 28, 39, 50, 60, 67, 80, and 84, each rated from 0 (no) to 1 (yes). If two or more “yes” answers are selected from the six sub-items of Item 84, the score of Item 84 will be counted as 1. A higher score of this subscale indicates more borderline personality traits and a score of 5 or higher is classified as borderline personality disorder. In the current sample, Cronbach’s α was 0.678.

#### Self-control

The Chinese revision of the Self-Control Scale (SCS) was used to assess the level of self-control in adolescents [38, 39]. The scale has 19 items, each rated on a 5-point Likert scale, ranging from 1 (strongly disagree) to 5 (strongly agree). A higher score indicates better self-control ability in adolescents. In the current sample, Cronbach’s α was 0.890.

#### Adolescent depressive symptoms

The Children’s Depression Inventory (CDI) was used to assess adolescent depressive symptoms [40]. It has been normalized in China with high reliability and validity, the Cronbach’s α coefficient of which was 0.88 and the criterion-related validity coefficient of which was 0.44 [41].The scale has 27 items, each rated on a 3-point scale (0–2) to measure depressive symptoms in adolescents between 14 and 18 years old. A higher score indicates more severe depressive symptoms and a score of 19 or higher is classified as depression. In the current sample, Cronbach’s α was 0.877.

### Statistical analysis

All data were analyzed using SPSS 25.0. Descriptive statistics were reported as mean (*M*) ± standard (*SD*) or frequency (percentage). For the primary analysis, Spearman correlation analyses were performed to examine the associations among childhood trauma, borderline personality traits, self-control and depressive symptoms. For the chain mediating effect analysis, SPSS PROCESS macro 3.3 software was used to examine the mediating role of borderline personality traits and self-control between childhood trauma and adolescent depressive symptoms. Gender and age were taken as covariates. Model 6 in PROCESS was applied for the chain mediators. Indirect effects were estimated through a bias-corrected bootstrapping procedure. A significant mediation effect was indicated if the 95% confidence interval (CI) did not encompass 0. The significance value was set at *p* < 0.05 (two-tailed) in this study.

## Results

### Demographic and descriptive analysis

Out of 2,664 valid questionnaires, the prevalence of depression in adolescents (CDI ≥ 19) was 21.17%. The prevalence of borderline personality disorder in adolescents (the score of borderline personality ≥ 5) was 12.00%. The sample of adolescents was aged from 14 to 18 years (*M* (age) = 16.54, *SD* = 0.95). Table 1 shows the descriptive statistics and scores of childhood trauma, borderline personality traits, self-control and depressive symptoms in adolescents.

**Table 1:** Descriptive statistics and scores of childhood trauma, borderline personality traits, self-control and depressive symptoms

Variables	M ± SD / *n* (%)
Age (years)	16.54 ± 0.95
Gender	
Boys	1289(48.39)
Girls	1375(51.61)
Childhood trauma (CTQ-SF)	34.31 ± 8.07
Borderline personality traits	2.21 ± 1.78
Self-control (SCS)	58.86 ± 11.10
Adolescent depression (CDI)	13.35 ± 7.27

### Correlation analysis

Table 2 shows correlations among childhood trauma, borderline personality traits, self-control and depressive symptoms. Childhood trauma (*r* = 0.50, *p* < 0.001) and borderline personality traits (*r* = 0.60, *p* < 0.001) were positively correlated with adolescent depressive symptoms, while self-control was negatively correlated with depressive symptoms (*r* = − 0.50, *p* < 0.001). All participants who experienced childhood trauma, and had borderline personality traits and self-control showed moderate to strong correlation with depressive symptoms (|*r*| > 0.50, *p* < 0.001).

**Table 2:** Correlations between childhood trauma, borderline personality traits, self-control and depressive symptoms

	1	2	3	4	5
1. Childhood trauma	−				
2. Borderline personality traits	0.34^***^	−			
3. Self-control	−0.23^***^	−0.45^***^	−		
4. Depressive symptoms	0.50^***^	0.60^***^	−0.50^***^	−	

*Note* ***p < 0.01

### The mediating effects analysis

**Fig. 1:**
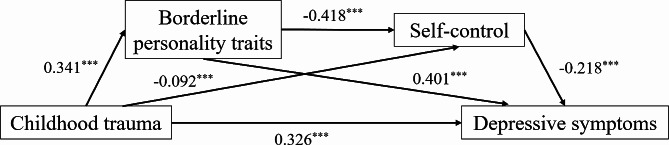
The chain mediation model for childhood trauma, borderline personality traits, self-control, and depressive symptoms. ^***^p < 0.001

Childhood trauma, borderline personality traits, self-control and depressive symptoms were significantly correlated, meeting the requirements for further mediation analysis between childhood trauma and depressive symptoms [42]. Table 3 shows significant mediating effects of borderline personality traits and self-control on the relationship between childhood trauma and depressive symptoms (effect value: 0.034, 95%CI = [0.028, 0.042]). The model fits well as shown in Fig. 1. Childhood trauma directly affected depressive symptoms, with a direct effect of 0.278 and an effect size of 61.78%. Childhood trauma indirectly affected depressive symptoms through borderline personality traits, with a mediating effect of 0.116 and an effect size of 25.78%. Childhood trauma also indirectly affected depressive symptoms through self-control, with a mediating effect of 0.022 and an effect size of 4.89%. Furthermore, childhood trauma indirectly affected depressive symptoms by borderline personality traits and then self-control. Together, they play a mediating role between childhood trauma and depressive symptoms, with a mediating effect value of 0.034 and an effect size of 7.56%. In this model, the mediating effect of borderline personality traits was stronger.

**Table 3:** Bootstrap analysis of the significance test of the mediating effect

Path	Effect	Effect size (%)	SE	Bias-corrected 95% CI	
				Lower	Upper
Total effects	0.450		0.015	0.421	0.480
Direct effects	0.278	61.78%	0.013	0.253	0.304
Childhood trauma → Borderline personality traits → Depressive symptoms	0.116	25.78%	0.010	0.098	0.137
Childhood trauma → Self-control → Depressive symptoms	0.022	4.89%	0.005	0.012	0.032
Childhood trauma → Borderline personality traits→Self-control → Depressive symptoms	0.034	7.56%	0.004	0.028	0.042

*Note* CI: confidence interval; effect size, %: the ratio of the effect to total effect; *SE*: standard error

## Discussion

The main findings of this study were that childhood trauma and borderline personality traits were positive predictors of depressive symptoms in adolescents, while self-control was a protective factor of depressive symptoms in adolescents. Borderline personality traits and self-control have a mediating effect on the relationship between childhood trauma and adolescent depressive symptoms respectively. Borderline personality traits and self-control play a chain mediating role in the relationship between childhood trauma and depressive symptoms in adolescents.

This study shows childhood trauma can positively predict depressive symptoms, in line with previous research [43], probably because negative childhood experiences damaged mental resilience, increasing the risk of depression [44]. Childhood trauma also increases adolescents’ risk of self-harm behaviors and even suicide attempts [45]. According to the hopelessness theory of depression, childhood trauma especially emotional abuse and neglect leaves individuals particularly vulnerable to developing a negative cognitive style, which in turn increases the risk for depression [46]. Parents should strive to minimize traumatic experiences during the upbringing of their children. By doing so, children can thrive in a nurturing and warm educational environment, facilitating their social development.

Our findings demonstrate that borderline personality traits act as a mediating factor in the correlation between childhood trauma and depressive symptoms in adolescents. Borderline personality traits were associated with an increased risk of depressive symptoms in adolescents, in line with previous studies [43, 47]. Borderline personality disorder further predicted the development and relapses of major depression disorder [48]. Borderline personality disorder and depression were likely to be comorbid, and such patients were more likely to have histories of suicide attempts and self-mutilation [49]. The association between borderline personality disorder and major depression disorder was partially explained by shared pathological personality traits, which may be underpinned by disturbances of cognitive control [32]. Cognitive dysfunction is associated with childhood trauma in mood disorders [50]. Childhood trauma can induce alterations in the hypothalamic-pituitary-adrenal axis, neurotransmission, endogenous opioid systems, and neural plasticity, which increase an individual’s susceptibility to developing borderline personality disorder [51]. Borderline personality disorder is reflected in the intense emotionality and impulsive behaviors that are often self-destructive. People with borderline personality traits also possess extreme sensitivity to perceived interpersonal slights, manifested as unstable low self-esteem and feelings of rejection that were labile in response to daily interpersonal stress. All these traits contributed to the development of depression [49].

The mediating role of self-control between childhood trauma and depressive symptoms in adolescents has been found in this study. Self-control was negatively related to depressive symptoms in adolescents. As reported in previous literature, individuals with high self-control exhibit lower levels of depression compared to those with low self-control [52]. It was probably because the ability of self-control in adolescents promoted the formation of positive adaptation strategies and released the stress they perceived, which reduced their depressive symptoms [30, 53]. Meanwhile, low self-control ability makes it more difficult for people to regulate their emotions and behaviors, increases the risk of losing control of their emotions and behaviors, and makes them more likely to develop depressive symptoms [54]. Childhood trauma can influence adolescent depressive symptoms through the depletion of sensitivity. Moreover, the mediating effect of depleted sensitivity is also moderated by the current level of self-control in adolescents [55]. Furthermore, Childhood trauma affects self-control through the mediating role of depletion sensitivity [28]. Childhood trauma, by reducing self-esteem, diminishes adolescents’ self-control, ultimately increasing adolescent aggression [56]. Low self-esteem predicts depression and aggression is an obvious symptom of adolescent depression [57, 58].

The chain mediating effect of borderline personality traits and self-control between childhood trauma and depressive symptoms indicated a series of effects of traumatic experiences on adolescents. In this process, first of all, childhood trauma has a significant impact on the formation of borderline personality traits, which results from the negative rearing of caregivers [22]. In terms of functional magnetic resonance imaging of the brain, childhood trauma was associated with the functional connectivity enhancement between the left insula and limbic system-prefrontal circuit [59], which meant poorer emotion regulation ability and higher likelihood of borderline personality traits [60, 61]. Emotion regulation and impulsivity control interact with the mechanisms of BPD [62], BPD is closely linked to self-control capacity [63]. Self-control refers to the capacity to regulate one’s impulses, emotions, or behaviors to achieve long-term goals [64–66], which serves as the internalized psychological foundation for coping strategies. From the perspective of defense mechanisms in psychodynamics [67], childhood trauma was associated with the formation of immature defense mechanisms [68], such as avoidance, regression and dissociation. Negative defensive styles explained the level of psychological suffering caused by childhood trauma [69], which was related to the high risk of borderline personality disorder [70]. In addition, low self-control receives more negative evaluation in Chinese culture [71, 72], and consciously suppressing and reducing emotions in adolescents aligns more with the needs of Chinese culture [73]. In this context, the loss of control and negative social evaluation further exacerbated depressive symptoms in adolescents [54].

The key strength of this study is the comprehensive exploration of the progression from childhood trauma to depressive symptoms in adolescents. Childhood trauma not only has short-term direct effects on adolescents, such as altering their emotional states, but also has long-term indirect effects on their personality traits and abilities, such as forming borderline personality traits and reducing self-control. These negative effects would finally increase negative self-perceptions, which leads to suicidal ideation in adolescents. The results help us understand the effect of childhood trauma and the risk of depressive symptoms from the perspective of personality traits.

There are several limitations to this study. Firstly, this study was a cross-sectional rather than a longitudinal-up study, which means that the childhood trauma of adolescents could only be measured through the memory of adolescents. Secondly, our data were obtained only in Henan, so that the findings may not extrapolate to other parts of China. Thirdly, the influence of genetic factors and economic level was not excluded in this study.

## Conclusion

The results of our study showed that childhood trauma was positively related to depressive symptoms due to the formation of borderline personality traits and the reduction of self-control. These findings call on parents to attach importance to the impact of negative experiences on their children, the formation of personality traits and the warning signs of depressive symptoms in adolescents. Enhancing teenagers’ self-control abilities has emerged as a crucial adaptive strategy. Educators and clinicians can analyze the causes of depressive symptoms in adolescents from the influence of the external environment during adolescents’ developmental phases, such as the personality traits and coping strategies shaped by traumatic experiences.

## Data Availability

All data generated or analyzed during this study are not publicly available due to the privacy of the participants identities. The data used to support the findings of this study are available from the corresponding author upon request.
